# Commercial Rabbit Farming and Poverty in Urban and Peri-Urban Kenya

**DOI:** 10.3389/fvets.2020.00353

**Published:** 2020-06-19

**Authors:** Chrispinus Mutsami, Stephen Karl

**Affiliations:** ^1^IGAD Climate Prediction and Applications Center, Nairobi, Kenya; ^2^International Livestock Research Institute, Nairobi, Kenya

**Keywords:** commercialization, multidimensional poverty, rabbit, Kenya, welfare, control function

## Abstract

Research has shown that agricultural commercialization is an effective way of boosting farmers' welfare. Is this true for urban or peri-urban farmers? We attempt to answer this question by assessing the effects of rabbit commercialization on multidimensional poverty among urban farmers in Kenya. While previous studies have analyzed commercialization in terms of crops, small livestock such as rabbit has received little attention. Additionally, most studies use income to capture poverty without considering other deprivations such as education, health and living standards. Here, we assess the effect of rabbit commercialization on multidimensional poverty among urban and peri-urban farmers. Data from 260 respondents is used. Findings show that rabbit commercialization is associated with a decrease in multidimensional poverty among urban and peri-urban farmers. This means that rabbit commercialization has a potential of improving living standards of urban poor. Other findings show that multidimensional poverty is positively associated with increase in education, access to credit, and reduced family sizes. Policy implication of our findings is that there is need to focus on promotion of commercialization among smallholder urban farmers through expansion of microfinance sector among urban dwellers to reduce financial market failures caused by inadequate access to financial services. Additionally, we recommend the promotion of training programs in different sectors such as rabbit farming. Urban dwellers with large households to be empowered to ensure all household members participate in income generating activities such as rabbit farming and commercialization.

## Introduction

Rabbit (*Oryctolagus cuniculus*) farming is one of the fastest growing micro-livestock enterprises in Kenya. Rabbit is preferred as a sustainable source of proteins in an era where climate change, population and changing meat consumption patterns are growing in developing countries (1). Additionally, rising per capita income, growing urbanization, and unfolding globalization are boosting the demand for high-value commodities including meat (2). Due to these fast socio-economic changes in the recent past, a rapid shift has taken place in the dietary habits in favor of sustainable sources of protein such as rabbit meat. Urban and peri-urban farmers in Kenya have started engaging in rabbit farming to satisfy the existing demand especially in urban areas.

Rabbit farming can be sustainable in developing countries due to the following reasons. First, rabbits can be raised on a grain free-diet. In a world of rising prices and increasing demand for grain, the ability to raise a good protein on garden forage is a plus in poor countries. Second, rabbits are characterized with fast growth rate, high fecundity, high feed conversion efficiency, and early maturity. With good husbandry, rabbits can produce above 40 kits per annum compared to one calf for cattle and up to two kids in goats (3). Third, rabbits are considered free from odor, noiseless, and can adapt in many ecosystems unlike many of the larger ruminants (4). Lastly, research shows that farmers in developing countries have started showing interest in information and communication technologies (4). Such technologies are used as marketing platforms where farmers and potential buyers meet. Such arrangements make it easy for farmers to sell their produce. For instance, there is an online platform in Kenya called *Mkulima Young* designed to transform smallholder farming by providing market access to farmers.

Initially, rabbit farming was done as a hobby or for subsistence purposes (5). In the recent past, rabbit production has been changing from non-commercialized to commercial one (6). Commercialization in this case means changing from subsistence to market-oriented farming. Commercialization in agriculture is associated with poverty reduction, income growth, and employment creation (7). Commercialization of farming also increases food supply in urban areas thus important in improving food security and nutrition (8). However, in the absence of any systematic study, there have been questions from the entrepreneurs, progressive farmers, and even researchers on the welfare outcomes—such as poverty—of commercial rabbit farming especially in urban and peri-urban areas. This paper, probably for the first time, is aimed at addressing issues related to commercialization of rabbit farming and the related effects on poverty among urban and peri-urban farmers in Kenya and to evolve a suitable policy framework for this promising sector of livestock.

We contribute to the existing literature in two ways. First, recently, we have witnessed an increased interest in urban farming by researchers and development partners. However, most of their focus has been on crops—especially kitchen gardening—and dairy farming (9). Small livestock have rarely been studied. Despite the role of rabbit farming in improving livelihoods, according to our knowledge, no study has been done to elicit their role in improving welfare of urban and peri-urban households. The second contribution relates to the measurement of commercialization and poverty. Most studies use income to capture poverty (8, 10). Income alone may not capture the multidimensional nature of poverty. The current study will contribute to the existing literature by considering other dimensions of poverty—such as education, income and living standard indicators.

## Materials and Methods

### Study Area

**Figure 1:**
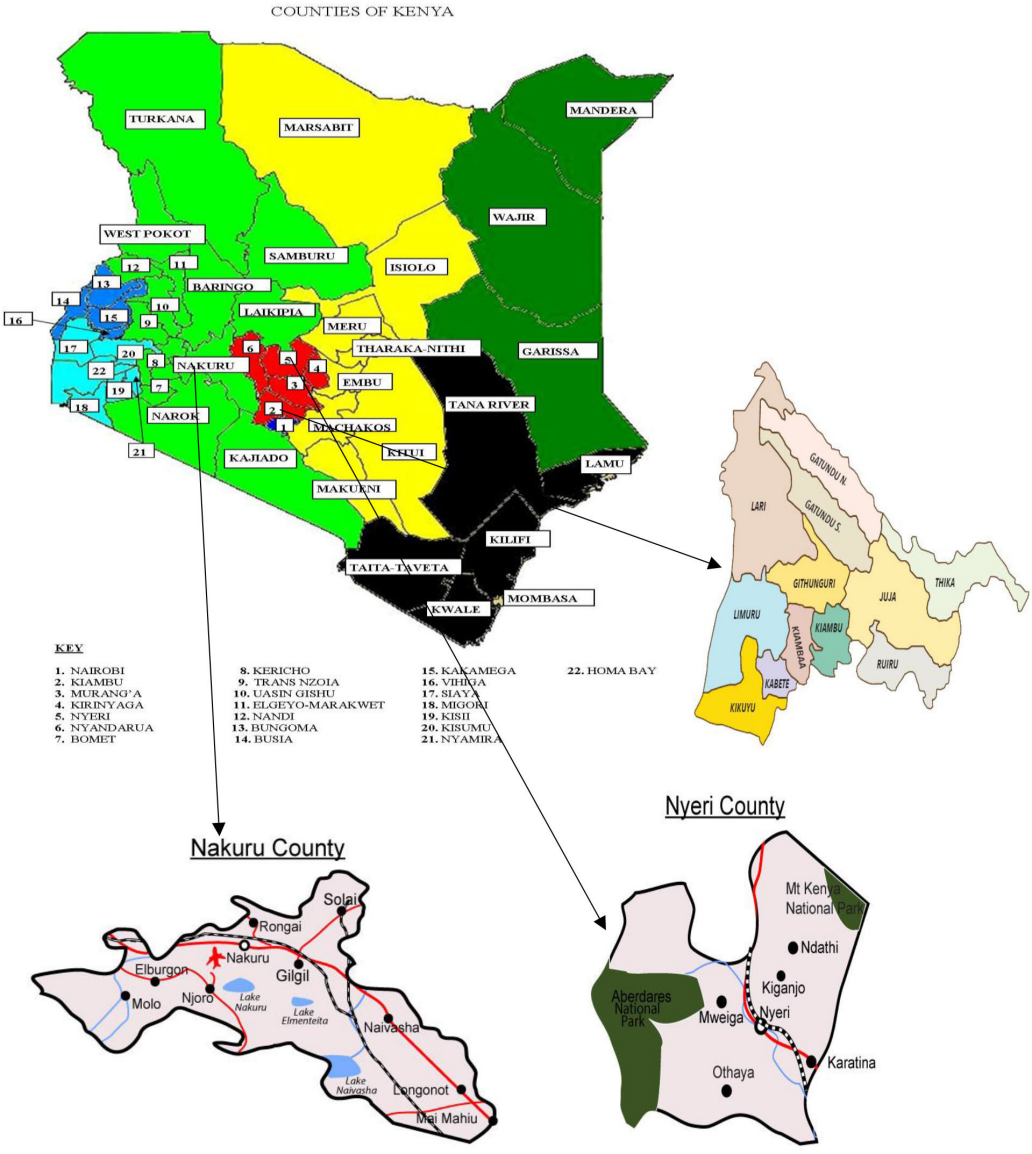
Map of Kenya showing Nakuru, Nyeri and Thika. Source: Kenya National Bureau of Statistics (KNBS) (11).

The data were collected from three major towns in Kenya—Thika (Kiambu County), Nakuru (Nakuru County), and Nyeri (Nyeri County). The three towns were selected based on their high number of rabbit producers and their proximity to Nairobi (Kenya's capital city) (Figure 1). The towns are rapidly urbanizing with population between 100,000 and 500,000. The study areas provide an excellent context of studying how commercialization of urban farming can influence welfare of households. All the three towns have favorable conditions for farming—such as bimodal type of rain whereby the long rains are received in the months of April, May, and August while the short rains are received between the month of October and December.

### Study Population

The towns are also characterized with several slum settlements that have grown in number. For instance, 47% of people in Nakuru live in informal settlements (11). The bulk of those residing in slums are employed in informal sector while the rest provide labor in the industries existing in the respective towns. Some of the households practice urban farming to supplement the income from non-farm activities. They keep small livestock such as chicken and rabbits while others practice crop farming—especially from hired farms outside the towns.

### Selection of Study Units

Data collection was conducted in July 2018. The sampling frame was from a list of farmers who had reported in 2012^1^ that they kept rabbits (12). Systematic random sampling, where we conducted an interview after every five farmers on the list, was employed. The sample size used in 2012 was 420. However, due to migration, shift in agricultural activities and difficulties in locating some farmers, we were able to re-interview 260 respondents.

### Data Collection

Face-to-face interviews with household heads, any household member above the age of 18 years and was responsible for the management and marketing of rabbit enterprise, were conducted by a team of well-trained and independent local enumerators. A carefully pre-tested structured questionnaire was used. The information captured included farm and household characteristics, rabbit production and marketing, a large range of institutional characteristics and asset ownership. Data on production and marketing were captured for a period of 12 months.

Key informant interviews were also conducted to collect information on rabbit marketing from a wide range of people for example, community leaders, representatives from Ministry of Agriculture, Livestock and Fisheries' in the sub-County, farmer/trader associations and managers from Kenya Rabbit Breeders Association. The interviews enabled getting first-hand information about rabbit enterprise in areas of study thus helped in validating our questionnaire.

### Measuring Key Variables

#### Measuring Rabbit Commercialization

Commercialization of rabbits was measured by computing the share of value of rabbits sold over a period of 12 months prior to the survey (Equation 1). And, the value of commercialization index ranged between zero and one.

(1) $$\begin{array}{l} C_{i}=\frac{\sum_{r=1}^{r}P_{r}Q_{ir}}{\sum_{R=1}^{R}P_{R}Q_{iR}} \end{array}$$

where *Q*_*ir*_ is the total number of rabbits *r* sold by household *i* evaluated at an average community level price *P*_*r*_. *Q*_*iR*_ is the total number of rabbits *R* produced by household *i* at an average community level price *P*_*R*_.

#### Measuring Multidimensional Poverty

**Figure 2:**
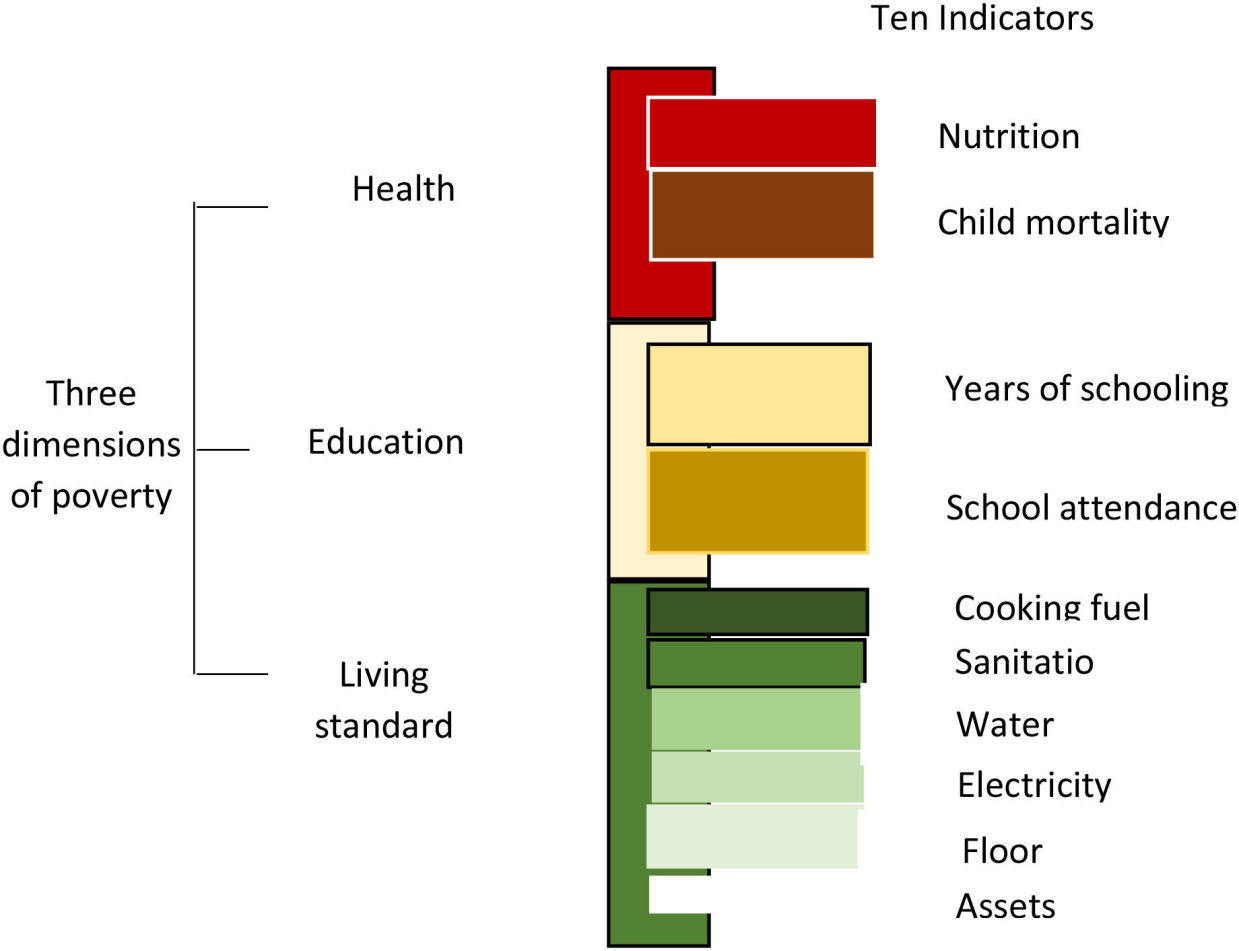
Dimensions of MPI. Source: Alkire and Santos (13).

The study uses multidimensional poverty index (MPI) to capture household's poverty. According to Alkire and Santos (13), the index is computed from a set of 10 deprivations across three dimensions: health, education, and standard of living (Figure 2). The procedure of constructing MPI entails defining the set of indicators which are to be considered in the multidimensional measure. Data for all indicators need to be available for the household. The next step involves setting the deprivation cut-offs for each indicator, namely the level of achievement (normatively) considered sufficient in order to be non-deprived in each indicator. The cut-off is used to determine whether each household is deprived or not in each indicator.

In this study, we closely follow the procedure as described by Alkire and Santos (13). However, we adjust some indicators to fit in the available data. For example, we use number of times family members fell sick within the last 12 months to measure health. Additionally, instead of cemented floor, we used amount of money paid as rent on monthly basis. This is because most of the households in some of the towns do not own houses. Instead they are tenants. All the MPI indicators are dummy variables with values of one or zero. Using weighting scores of 1/6 for education and health indicators and 1/18 for living standard indicators, we calculated MPI for each household. We first constructed household deprivation score, continuous variable with values ranging between zero and one, calculated by adding values for all the indicators. Using the household deprivation score, we created a dummy variable with one indicating a household's deprivation score being greater than a threshold of 0.33 and zero otherwise (13) (Table 1).

**Table 1:** Dimensions and indicators used to construct multidimensional index.

**Indicator**	**Description**
Education	Years of schooling of the household head
	Household has school-aged child not attending school
Health	Number of times household members fell sick in the last 12 months
Living standards	Access to safe drinking water
	Monthly rent paid
	Cooking with wood or charcoal
	Owns radio, television set, mobile phone, bikecycle, motorcycle, refrigerator
	Connected to electricity

*Source: Alkire and Santos (13) and Ogutu and Qaim (8)*.

There are several reasons for choosing MPI over other measures of poverty such as income and “dollar-a-day.” The approach has the following advantages: First, it produces an MPI measure that is robust with either ordinal or cardinal variables. Second, it fulfills the dimensional monotonicity condition. Third, it is decomposable by population subgroups. Fourth, it can be broken down by indicator, thus facilitating a deeper understanding of poverty structures and impact mechanisms. Finally, it provides absolute poverty levels (in intensity terms) that allow comparison of poverty across different settings (8).

Other variables—used as controls—were analyzed. Specifically, we assessed how socioeconomic factors, such as age, gender, household size, access to credit, group membership, and income influence poverty among livestock farmers.

### Empirical Strategy

We examine the effect of rabbit commercialization on multidimensional poverty using the following regression,

(2) $$\begin{array}{l} y_{i}=\beta_{0}+\beta_{1}C_{i}+\beta_{2}{\text{W}}_{\text{i}}+\varepsilon_{i} \end{array}$$

where *y*_*i*_ represents MPI, *C*_*i*_ is the commercialization index for each household, **W**_**i**_ is a vector of control variables i.e., household characteristics. The coefficient β_1_ represents the effect of commercialization on poverty and ε_*i*_ is random error term. Due to binary nature of *y*_*i*_, we use logit estimator to run Equation (2).

A positive coefficient β_1_ is expected thus a positive relationship between commercialization and MPI. A positive or an increase in MPI means a reduction in poverty levels among urban or peri-urban households. However, the level of rabbit commercialization is potentially endogenous meaning that we might have a correlation tween commercialization and the error term. The potential endogeneity is associated with biased and inconsistent β_1_. Some of the sources of endogeneity include unobserved heterogeneity, reverse causality, or measurement error (8).

To address the issue of endogeneity, the study employs control function (CF) method as described in (13, 14). The CF approach entails estimating residuals from a first-stage regression model of the determinants of commercialization, having at least one valid instrument. Since *C*_*i*_ is a censored variable (fraction bounded between zero and one), we estimate the first-stage regression using a fractional logit. The predicted residuals are then included in logit model as shown in Equation (2).

This study used instrumental variable in the first stage to come up with predicted residuals. Instruments in this case are supposed to be correlated with commercialization but not poverty (dependent variable). We used one instrumental variable i.e., number of households in the village who had contracts with supermarkets excluding the household being interviewed. The choice of the instrument is inspired by the recent literature on supermarkets and contracts (15, 16). It is evident that households who participate in supermarket contracts benefit more hence they are encouraged to produce more output that can be sold. Participation in supermarket contracts can positively influence commercialization of other rabbit farmers through social learning—peer to peer learning. Farmers in the same village are likely to learn from each other on the benefits of selling to local supermarkets. We tested for possible correlation between the instrument and MPI (poverty indicator). The correlation coefficient was found to be insignificant and weak indicating validity of our instrument.

## Results and Discussion

### Descriptive Statistics

A summary of household descriptive statistics is presented in Table 2. The average proportion of households who sell their rabbits is 52%. A previous study by Serem et al. (12) showed only 36% of rabbit farmers sold their rabbits in 2013. The significant increase in the proportion of farmers selling their rabbits may be attributed to establishment of rabbit value addition factories (such as RABAK in Thika town) and continued promotion of eating healthy diet by government and non-governmental organizations. In terms of multidimensional poverty, 54% of the households have a deprivation score of more than 0.33 hence classified as poor out of which 61% are headed by females. Majority of the households are male headed with an average household size of 5 who live near markets with an average annual income of Ksh. 330,600. In addition, only 31% reported to have membership in groups which can plausibly explain the low rate of credit access. On average, each village recorded 15 households who were selling rabbits to supermarkets. The average price for a mature rabbit was found to be Ksh. 525. The high price may be attributed to the high demand of rabbit white meat.

**Table 2:** Summary of household descriptive statistics.

**Variables**	**Mean**	**Std. Dev**.
Commercialization (share)	0.52	0.49
Multidimensional poverty (dummy)	0.46	0.34
Female-headed household	0.28	0.38
Female-poor	0.61	0.73
Age of household head (years)	47	12.31
Education of head (years)	9.88	4.09
Household size	5.14	3.21
Household size squared	32.68	24.79
Household income (1,000/year)	330.56	274.1
Group membership	0.31	0.46
Access to credit	0.37	0.48
Sellers in supermarkets in the village	15.62	36.33
Productive assets (1,000 Ksh)	12.09	11.17
Distance to the nearest market	1.77	2.25
Feed expenditure (1,000 Ksh/rabbit)	0.81	0.53
Average price	525	119.54
Region, Nakuru	0.39	0.45
Region, Thika	0.45	0.23
Region, Nyeri	0.16	0.41

*Source: Author's survey (2018)*.

**Figure 3:**
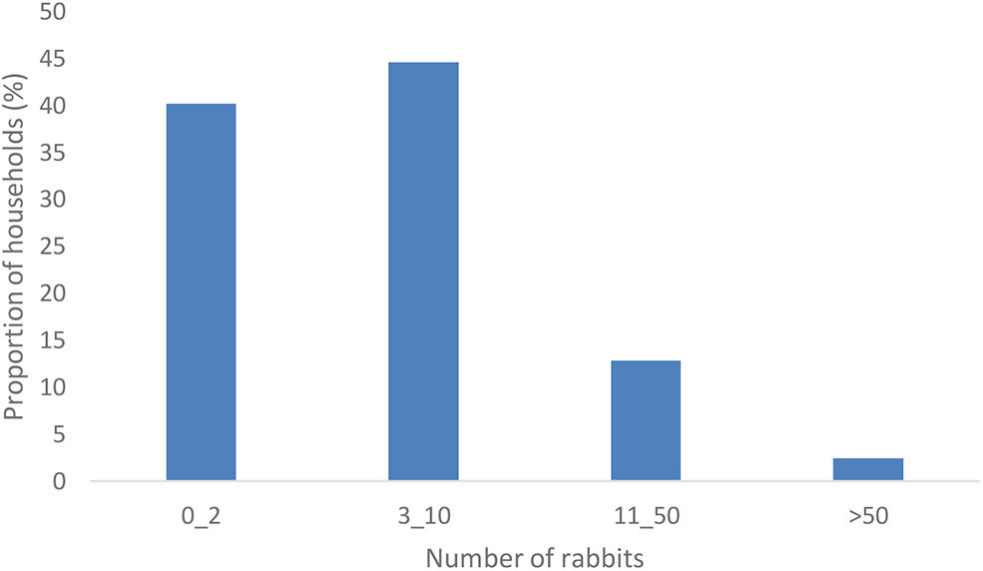
Average number of rabbits kept.

Figure 3 presents the number of rabbits kept by households. Of the total farmers interviewed, 40% kept utmost two rabbits. According to Oseni et al. (17), such farmers are classified as ultra-smallscale. About 45% reported that they kept 3–10 (smallscale), 13% kept 11–50 (medium scale) while 3% kept above 50 rabbits largescale).

**Figure 4:**
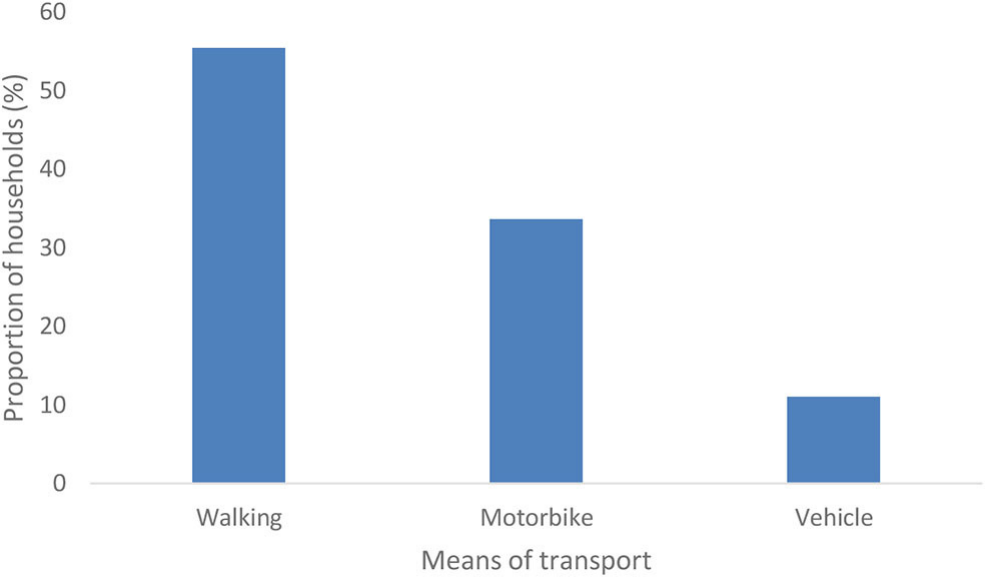
Means of transporting rabbits to the market.

We also investigated the means of transporting rabbits to the market (Figure 4). Most (55%) of the farmers walk to the market to sell their rabbits. This may be linked to the proximity of markets in study areas. Additionally, some farmers said they sell their rabbits to their neighbors which does not require motorized means of transport. This finding corroborates with that of Bett et al. (18) who found that walking was the major form of transport for those who were handling improved chicken in Kenya. Some (34%) of the rabbit actors used motorbikes—popularly known as *bodaboda*—to transport their rabbits to the market. Pick-up trucks were used for transportation especially for those who had more than 50 rabbits. It was noted that there were no specialized rabbit transporters in all the study areas.

### Effect of Commercialization on Multidimensional Poverty

The effects of commercialization on multidimensional poverty is shown in Table 3. Estimates for two models, logit and CF, are provided. As discussed above, the CF model involved two stages where residuals were predicted in the first stage. The CF results show a statistically insignificant residuals thus we fail to reject the null hypothesis of commercialization being exogeneous. The estimates in both approaches are almost similar. For the interpretation purposes, we concentrate on estimates without residuals as they are considered to be more efficient.

**Table 3:** Effect of commercialization on multidimensional poverty.

**Variables**	**Logit model**	**CF**
Commercialization	−0.131^***^ (0.009)	−0.133 ^***^ (0.041)
Female-headed household	−0.023 (0.021)	−0.025 (0.022)
Age of household head (years)	−0.011 (0.001)	−0.011 (0.002)
Education of head (years)	−0.060^***^ (0.003)	−0.061^***^ (0.005)
Household size	0.017^**^ (0.008)	0.018 (0.006)
Household size squared	−0.001^*^ (0.002)	−0.001^*^ (0.002)
Non-farm income (1,000/year)	−0.012 (0.018)	−0.014 (0.013)
Group membership	−0.017 (0.011)	−0.018 (0.009)
Access to credit	−0.118^***^ (0.017)	−0.116 (0.013)
Productive assets (1,000 Ksh)	−0.002 (0.006)	−0.002 (0.006)
Distance to the nearest market	−0.009 (0.012)	−0.007 (0.011)
Feed expenditure (1,000 Ksh/rabbit)	0.015 (0.041)	0.022 (0.048)
Region dummies	Yes	Yes
Residual from first stage		−0.031 (0.021)
R-squared	0.21	0.218

*** *p < 0.01*,

** *p < 0.05*,

* *p < 0.1*.

*Standard errors are in parentheses and clustered at the household level*.

Table 3 shows that rabbit commercialization is associated with a negative and significant effect on poverty. Controlling for other factors, rabbit commercialization is likely to reduce the probability of being poor by 13%. These finding suggests that participation in markets by rabbit farmers is likely to reduce their poverty levels.

Our findings are to some extent in line with other studies on the effect of commercialization on poverty on other agricultural produce other than rabbit. For example, for smallholder bean producers in Western Kenya (8) conclude that agricultural commercialization results in reduction of multidimensional poverty. A few other studies also provided evidence that commercialization had a positive effect in poverty reduction among African smallholders (19–21). However, most of these studies used income to capture level of poverty. Income alone may overestimate or underestimate the real poverty when other factors, such as health, education and living standards, are not considered.

Some of our control factors have significant effect on poverty status of rabbit farmers. For instance, household size is associated with 1.7% increase in multidimensional poverty. This is in line with the literature (22, 23) where it is noted that larger families tend to be poorer in developing countries. Following micro-economic arguments, in Kenya, children are considered as an essential part of the household's work force to generate household income, and as insurance against old age. However, a high number of children and their participation in household production are likely to impede investment in their human capital (i.e., education and health), maintaining the low-income status of the household, and thereby creating or perpetuating a poverty-fertility trap.

Access to credit is associated with 11.8% reduction in poverty among rabbit farmers. Negative effect of access to credit on poverty is associated with availability of capital which can be invested in rabbit farming hence increasing production and productivity. The availability of financial institutions helps to reduce market failures caused by inadequate access to and provision of financial services (21). Our finding differs with that of Imai et al. (24) where it was reported that access to credit had no effect on poverty in Northern region of Thailand. Lack of significant effect was attributed to poor targeting of the credit intervention.

Increase in level of education and the number of years one stays in school was associated with 6% reduction in multidimensional poverty among rabbit farmers in urban and peri-urban Kenya. Education increases the stock of human capital, which in turn increases labor productivity and/or wages which can be plowed back in rabbit farming. Since labor is by far the most important asset of the poor, increasing the education of the poor will tend to reduce poverty. In fact, there appears to be a vicious cycle of poverty in that low education leads to poverty and poverty leads to low education.

## Conclusion

Findings show that rabbit commercialization is associated with a decrease in multidimensional poverty among urban and peri-urban farmers. This means that livestock commercialization has a potential of improving living standards of urban poor. Policy implication of this finding is that there is need to focus on promotion of commercialization among smallholder farmers through provision of necessary infrastructure such as roads and refrigerators and institutions such credit, inputs and output markets.

Additional findings show that household size increase multidimensional poverty. It, therefore, means that larger households are poorer than smaller ones. We recommend that such families should be empowered to ensure they participate in economic activities such as rabbit farming and commercialization. This will allow large families to earn income that can sustain their livelihoods. We also find that access to credit reduces multidimensional poverty. We argue that expansion of microfinance sector among urban dwellers could reduce financial market failures caused by inadequate access to financial services. Additionally, financial institutions should aim for sustainable financial services. For instance, credit lending institutions such as commercial banks and micro-finance institutions should therefore work toward providing affordable and accessible credit to rabbit farmers in order to improve their ability to cover costs associated with production and marketing of rabbits. Education was found to reduce poverty among households in urban areas. We recommend promotion of training programs in different sectors such as rabbit farming.

## Data Availability Statement

The datasets generated for this study are available on request to the corresponding author.

## Ethics Statement

The studies involving human participants were reviewed and approved by University of Nairobi Institutional Review Board. The patients/participants provided their written informed consent to participate in this study.

## Author Contributions

CM and SK: study design, data analysis, and manuscript development. CM: data collection. All authors: contributed to the article and approved the submitted version.

## Conflict of Interest

The authors declare that the research was conducted in the absence of any commercial or financial relationships that could be construed as a potential conflict of interest.

## Footnotes

^1^This was done under the project “Strategies to improve the rabbit value chain in Kenya” supported by the Kenya National Council for Science and Technology.
